# Anti-Disgust Cognitive Behavioral Therapy for Contamination-Based Obsessive Compulsive Disorder: A Randomized Controlled Clinical Trial

**DOI:** 10.3390/jcm11102875

**Published:** 2022-05-19

**Authors:** Behzad Salmani, Francesco Mancini, Jafar Hasani, Zahra Zanjani

**Affiliations:** 1 Department of Clinical Psychology, Faculty of Psychology and Educational Sciences, Kharazmi University, Tehran 31979-37551, Iran; hasanimehr57@khu.ac.ir; 2 Department of Human Sciences, Marconi University, 00193 Rome, Italy; f.mancini@unimarconi.it; 3 Department of Clinical Psychology, School of Medicine, Kashan University of Medical Sciences, Kashan 87159-73474, Iran; zanjani-z@kaums.ac.ir

**Keywords:** obsessive compulsive disorder, cognitive behavioral therapy, disgust, anti-disgust cognitive intervention

## Abstract

Background: Disgust is a strong and persistent emotion that frequently occurs during exposure-based treatments for contamination-based obsessive compulsive disorder (C-OCD). This study aimed to examine the efficacy of augmenting cognitive behavioral therapy (CBT) with a novel type of anti-disgust cognitive intervention in reducing the severity of OCD, disgust propensity/sensitivity, and refusal rate of exposure and response prevention, while simultaneously increasing acceptance of disgust. Materials and Methods: Fifty-five individuals with C-OCD (mean age 28.1 years, SD = 3.52; 77% female) were randomly assigned to 15 weekly sessions of anti-disgust plus CBT (AD-CBT) or CBT alone. They were evaluated for outcomes four times (pretreatment, prior to exposure and response prevention (ERP) sessions, posttreatment, and three-month follow-up), and mixed-design ANOVAs were used to analyze the data. Results: The findings indicated that when compared to CBT alone, AD-CBT significantly reduced OCD severity, disgust propensity/sensitivity, and concurrently increased disgust acceptance (*p* < 0.001). Additionally, engaging in an anti-disgust cognitive intervention was associated with lower ERP refusal rate (4% vs. 16%). The superiority of AD-CBT over CBT persisted through the three-month follow-up period. Conclusions: The current study suggests that supplementing CBT for C-OCD with an anti-disgust cognitive intervention significantly increased acceptance of disgust and decreased the refusal rate of ERP, OCD severity, and disgust-related factors.

## 1. Introduction

Obsessive compulsive disorder (OCD) is defined by unwanted internal events that result in negative emotional states and repetitive compulsions to alleviate these negative emotional states. This disorder is one of the most prevalent psychiatric disorders in Iran [1], and it has a negative effect on both individuals and their families’ quality of life [2,3,4]. Obsessive compulsive disorder (OCD) is classified into several subtypes, with contamination-based OCD (C-OCD) being the most common [5]. While anxiety is a significant emotion in OCD, an increasing number of studies have recently demonstrated that individuals with C-OCD frequently express disgust, especially when they feel contaminated [6,7].

The literature has established a strong correlation between disgust and C-OCD symptoms. In a clinical sample, Athey et al. [8] demonstrated that decreasing disgust propensity, or how easily one is disgusted, alleviates contamination and excessive washing symptoms. Additionally, Olatunji et al. [9] found that the severity of OCD could be predicted in youth with OCD based on their disgust proneness, even when the negative affect was controlled. Furthermore, disgust sensitivity, or how negatively one perceives disgust, has been linked to OCD symptoms [10]. Moreover, neuroscience research on the role of disgust in C-OCD has grown in popularity [11,12]. Nonetheless, an area of difficulty arises when individuals with contamination fears are exposed to disgusting stimuli as part of exposure-based treatments.

Exposure and response prevention (ERP) is considered the gold standard for treating OCD [13]. According to the behavioral model, ERP results from habituation and inhibitory learning [14,15]. In comparison to fear, some studies found that disgust had a slower and more transient habituation course during ERP in individuals with C-OCD [6,16,17]. Disgust appears to be more resistant to change during treatment than fear [18,19]. This may help explain why some individuals refuse to participate in ERP sessions or why ERP sessions have a relatively high dropout rate [17]. As a result, it is critical to identify and target factors contributing to increased disgust during the ERP. Given the increased disgust response in C-OCD individuals or non-clinical samples with severe OCD symptoms [20] and the OCD sample’s negative appraisal of emotion [21], one of the promising factors is disgust beliefs.

The emotion regulation literature has implicitly evidenced that OCD individuals interpret emotions negatively [21]. For example, Berman et al. [22] demonstrated that OCD individuals frequently struggle with emotion regulation difficulties, including an inability to accept intrusive emotions and a failure to engage in goal-directed behavior. Moreover, Ferreira et al. [23] observed that individuals with OCD had difficulties utilizing more adaptive strategies, such as reappraisal. Furthermore, in a non-clinical sample, higher scores on emotion regulation strategies, such as suppression and emotional clarity, were associated with OCD symptoms [24]. Therefore, individuals with C-OCD are likely to exhibit a distorted perception of disgust, resulting in psychopathology. Thus, the acceptance of negative mental experiences, which they refer to as *habitual acceptance*, is believed to be associated with improved psychological health [25].

Despite the critical nature of disgust, CBT and its behavioral component, ERP, do not adequately address biased disgust interpretations [26]. Furthermore, clinicians are encouraged to incorporate disgust-related components into exposure-based treatments for C-OCD to decrease the ERP refusal rate [6]. Although some studies proposed strategies such as counterconditioning to target disgust evaluations [16], these strategies primarily targeted short-lived acquitted disgust evaluations rather than long-term beliefs. Additionally, the dropout rate of ERP has not been sufficiently addressed in these studies [27]. Accordingly, Perdighe and Mancini [28] proposed new types of cognitive interventions to alter real biased cognitions (compared to acquitted ones), such as beliefs about disgust emotion, based on recent empirical studies [29]. Additionally, some of the underlying mechanisms of disgust beliefs, such as deontological guilt and magical thinking, were not addressed during standard OCD treatments [28,29]. Moreover, the challenging beliefs of patients with C-OCD about informative and biased cognitions about disgust have been emphasized in recent years [30]. Given the lower response rate observed in C-OCD individuals during ERP [19], we hypothesize that altering emotional beliefs about disgust may have additional benefits when ERP is used to treat C-OCD.

The current study used an anti-disgust cognitive intervention, as per Perdighe and Mancini [28], to address disgust-related beliefs. The study examined the effects of adding an anti-disgust cognitive intervention to traditional CBT for OCD on (1) decreasing disgust propensity/sensitivity, (2) increasing non-judgmental acceptance of emotions, (3) decreasing OCD severity, and (4) decreasing ERP dropout rate. To assess whether the effect of the intervention is influenced by a negative or positive affect, the present study attempted to control positive and negative affect along with depressive symptoms. It is hypothesized that challenging beliefs about disgust could reduce the severity of OCD, disgust propensity/severity, and the ERP dropout rate, while simultaneously increasing acceptance of negative mental experiences, such as disgust. Finally, the present study compared outcomes in four assessment phases (pretreatment, prior to ERP sessions, posttreatment, and three-month follow-up) between two groups that included either an anti-disgust cognitive intervention plus CBT (AD-CBT) or CBT alone.

## 2. Materials and Methods

### 2.1. Participants

Participants (aged 19 to 40) were recruited from OCD patients referred to three outpatient mental health centers in Tehran and Kashan, Iran. All participants who met the criteria for C-OCD were included in the study if they met the following criteria: (a) completed at least 12 years of education, (b) were at least 18 years old, (c) were not diagnosed with other subtypes of OCD as a primary diagnosis, (d) did not have any comorbid disorders, except for major depressive disorder (MDD) and anxiety disorders, (e) were on a stable dose of medication three months prior to the study’s start, and (f) signed an informed consent form. C-OCD was diagnosed using the Persian version of the Structural Clinical Interview for DSM-5-Research Version (SCID-5-RV), conducted by a clinical psychologist with a Ph.D. in clinical psychology [31]. The authors used the Yale–Brown Obsessive Compulsive Scale (Y-BOCS) checklist of obsessions and compulsions to ascertain the primary presenting type of OCD [32]. To achieve 95%, power analysis using G*Power indicated that a sample size of 48 participants would be required (α = 0.05), assuming a moderate effect size (0.25). A total sample size of 60 participants was recruited to account for potential attrition.

**Figure 1:**
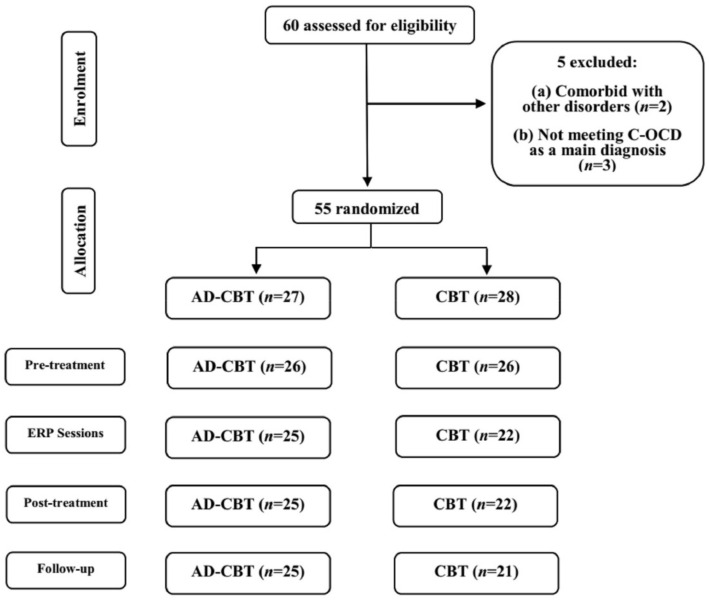
Consort flow diagrams of the study development. Note: AD-CBT = Anti-Disgust Cognitive Behavioral Therapy; CBT = Cognitive Behavioral Therapy; ERP = Exposure and Response Prevention; C-OCD = Contamination-Based Obsessive Compulsive Disorder.

Finally, 55 participants (77% female) who met the eligibility criteria were randomly assigned to receive either an AD-CBT (*n* = 27) or CBT alone (*n* = 28). Random assignment was performed by an automatic number generator. Three participants changed their medication dose or type following randomization, and their data were excluded from the final analysis. Patients in the two groups who took medication reported taking Citalopram 20 mg with or without Fluoxetine 10 mg (AD-CBT) or Citalopram 20 mg with or without Alprazolam 0.5 mg (CBT). In total, 38 (73%) of the 52 participants (AD-CBT: *n* = 26; CBT: *n* = 26) were diagnosed with C-OCD, and 14 were diagnosed with C-OCD comorbid with MDD or anxiety disorders. Additionally, five participants did not attend any ERP sessions, and one participant did not complete scales during the three-month follow-up period due to a new treatment regimen (Figure 1). It is critical to emphasize that none of the other patients received any new treatment during the three-month follow-up period. There were no differences in primary disorder, OCD severity, or demographic characteristics between participants who attended and participants who dropped out.

The study was approved by the ethical committee of Kharazmi University (IR.KHU.REC.1400.027) and registered in the Iranian Registry of Clinical Trials (IRCT20210914052475N1).

### 2.2. Therapists

Treatments sessions were provided by a clinical and a health psychologist with a Ph.D. in clinical and health psychology, respectively. The psychologists have over ten years of experience in treating OCD and anxiety disorders. Therapists who routinely delivered both AD-CBT and CBT were chosen to avoid bias in offering interventions. A clinical psychologist with over two decades of experience educating and treating individuals with OCD and anxiety disorders using CBT supervised the therapists. Overall, 379 and 346 sessions were administered by AD-CBT and CBT therapists, respectively.

### 2.3. Interventions

#### 2.3.1. Feasibility

To assess the feasibility of AD-CBT, five C-OCD individuals (age = 22–35; M = 27.1; SD = 3.91) underwent the intervention. Although the feasibility study lacked a control group, it demonstrated some ability to control confounding variables, such as therapist effects. Moreover, the feasibility study allowed the research team to discuss whether supplementing CBT with an anti-disgust cognitive intervention benefited the participants more than CBT alone. Each group received 15 weekly sessions. Table 1 summarizes the content of each session for both interventions.

#### 2.3.2. Cognitive Behavioral Therapy (CBT)

The cognitive behavioral therapy protocol used in this study was adapted from Kozak and Foa [33] and consisted of 15 weekly 90-min sessions. The first two sessions were dedicated to psychoeducation about the cognitive behavioral model. Participants also learned about avoidant behaviors in OCD and the importance of exposure and not responding to it with compulsions during sessions 1 and 2 [26]. Individuals were instructed to continue and rehearse what they had learned during psychoeducation during the third and fourth sessions. The therapist and participants collaborated to create an exposure hierarchy list, and the therapist encouraged participants to encounter anxiety/disgust-provoking situations in order of the hierarchy list over the subsequent ten sessions. The therapist weighted participants’ improvements following each ERP session using the cognitive behavioral model. The final session educated participants about relapse risk factors.

#### 2.3.3. Anti-Disgust Cognitive Intervention

Two 90-min sessions on anti-disgust cognitive intervention were adopted from the Perdighe and Mancini intervention [28] (sessions 3 and 4 on AD-CBT) that directly addressed disgust in the C-OCD sample. Participants were educated about the disgust function, the relationship between disgust and guilty feelings, and some of the underlying mechanisms of disgust, such as magical thinking, during session 3. The therapist then demonstrated the role of disgust in OCD by introducing the *Lady Macbeth* effect, a tendency to wash one’s own hand following a feeling of guilt, followed by a sense of relief. The therapist connected this effect to the imprecise and biased function of disgust in OCD during session 4. The therapist then discussed disgust’s physical and moral protective role in everyday life and shared some examples to help normalize disgusting feelings. Furthermore, the therapist discussed the non-threatening nature of intense disgust in C-OCD.

Finally, the therapist provided several instructive examples to help distinguish between the sensation of disgust and actual contamination. For instance, patients discovered that certain objects are highly contaminated but do not appear to be disgusting (e.g., a *mercury drop*) and vice versa (e.g., a *sterilized dead cockroach*). In general, the anti-disgust cognitive intervention is used to question the utility and accuracy of disgust-related beliefs. Perdighe and Mancini [28] (pp. 201–220) previously presented a detailed version of the anti-disgust cognitive intervention.

Participants in AD-CBT and CBT were instructed to apply what they learned in therapy sessions to situations involving disgusting stimuli as homework Additionally, they were informed that their therapist would assist them via telephone once a week if they encountered difficulties completing their homework. Finally, participants received a written version of treatment instructions that included components of AD-CBT or CBT to assist them if OCD symptoms relapsed. Both groups received essentially identical relapse prevention instructions as the relapse prevention program [34], except that the AD-CBT group was instructed to emphasize the role of disgust in a relapse of OCD symptoms.

### 2.4. Measures

#### 2.4.1. Demographic Characteristics

A six-item questionnaire was used to ascertain demographic characteristics. Moreover, pharmacologic information was gathered via three questions regarding the type of medication, its duration, and the number of doses taken by each participant.

#### 2.4.2. Structural Clinical Interview for the DSM-5-Research Version in Persian (SCID-5-RV)

The SCID-5-RV is a structural clinical-interview-based diagnostic tool based on DSM-5 criteria for psychiatric disorders [2]. The SCID-5-RV is intended for use by individuals over the age of 18. Shankman et al. [35] established the SCID-5-RV’s validity and reliability for diagnosing psychiatric disorders, such as OCD. SCID-5-RV takes 45–90 min to complete a clinical interview. Additionally, Mohammadkhani et al. [31] translated the measure and found that the Persian version of SCID-5-RV has acceptable psychometric properties, including internal consistency (0.95–0.99), test–retest reliability (0.60–0.79), and Kappa reliability (0.57–0.72).

#### 2.4.3. Disgust Propensity and Sensitivity Scale-Revised (DPSS-R)

The DPSS-R is a self-report scale comprising 16 items developed by van Overveld et al. [36] to assess disgust propensity and sensitivity. Participants respond on a five-point Likert scale ranging from one (“Never”) to five (“Always”; range 16–80). Preliminary studies have demonstrated that the scale has sufficient reliability. The alpha coefficients for the disgust propensity and disgust sensitivity subscales were 0.78 and 0.77, respectively. Furthermore, the DPSS-R has an acceptable level of content validity [36]. According to Zanjani et al. [37], the divergent validity and reliability of the Persian version of the DPSS-R make it appropriate for use in an Iranian sample. The authors of this study were interested in disgust as a unified concept. As a result, they combined the disgust sensitivity (DS) and propensity to disgust (DP) subscales. Cronbach’s alpha for the DPSS research was calculated to be 0.78 in the current study.

#### 2.4.4. Five Facets Mindfulness Questionnaire (FFMQ)

Baer et al. [38] developed the Five Facets Mindfulness Questionnaire (FFMQ) to assess the acceptance of internal experience. The questionnaire measures five aspects of mindfulness, including observing, describing, acting with awareness, non-judging of inner experience, and non-reactivity to inner experience. As one of the study’s aims was acceptance of the emotions and thoughts, “non-judging” and “non-reactivity” subscales were utilized. As per Baer et al. [38], both subscales are concerned with avoiding reappraisal of internal experiences and excessive reaction to them, which results in acceptance. In the current study, we referred to these subscales as the “Acceptance Scale (AS).” The FFMQ consists of 39 items, while each of the two subscales listed above contains 15 items (8 items assess non-judging and 7 items measure non-reactivity). Both FFMQ and AS were rated on a 5-point Likert scale ranging from 1 (never or very rarely true) to 5 (very often or always true). The internal consistency of the FFMQ subscales ranged from 0.75 (for non-reactivity) to 0.91 (for describing). Additionally, the measure’s reliability and incremental validity have been established in previous studies [38,39]. Moreover, Tamannaeifar et al. [40] asserted that the Persian version of the scale met all psychometric requirements in an Iranian sample. Internal consistency was estimated to be 0.79 for the “non-judging” and “non-reactivity” subscales.

#### 2.4.5. Yale–Brown Obsessive Compulsive Scale (Y-BOCS)

The Y-BOCS is a three-part semi-structured interview developed by Goodman et al. [32]. The first and second parts of the scale contain a checklist of obsessions and compulsions. The third section of the scale rates OCD severity. This section is divided into two sections that assess five different aspects of obsessions and compulsions [41]. Each item is rated on a 5-degree Likert scale from 0 to 4; the sum of the scores ranges from 0 to 40. Numerous studies have established the internal consistency, inter-rater reliability, and test–retest reliability of Y-BOCS in clinical and non-clinical samples [32,42,43]. According to Rajezi Esfahani et al. [44], the Persian version of the Y-BOCS is a valid and reliable instrument for assessing the severity of OCD. Internal consistency of Y-BOCS was well estimated in this study (Cronbach’s alpha = 0.81).

#### 2.4.6. Positive and Negative Affect Schedule (PANAS)

The authors used the PANAS to assess overall mood and affect as a trait to isolate positive and negative affect influences. Watson et al. [45] developed the PANAS, a 20-item measure divided into two subscales: positive affect (10 items) and negative affect (10 items). Respondents indicate how much each item applies to them on a 5-point Likert scale ranging from 1 (very slightly or not at all) to 5 (extremely). Furthermore, the Persian version of PANAS is shown to have acceptable psychometric properties in a clinical sample [46]. We used the total PANAS score in this study, including both positive and negative affects. Cronbach’s alpha coefficients for the positive and negative subscales were 0.74 and 0.76, respectively.

#### 2.4.7. Beck Depression Inventory (BDI-II)

The Beck Depression Inventory-II (BDI-II) is a widely used 21-item self-report scale used to determine the severity of depression in the preceding two weeks [47]. Respondents rate items on a 4-point scale ranging from 0 to 3. Depression is classified as minimal (0–13), mild (14–19), moderate (20–28), or severe (29–63). Dabson and Mohammad-Khani [48] determined that the Persian version of the BDI-II was a psychometrically valid measure in a clinical sample. The internal consistency of the BDI-II was acceptable in this study (Cronbach’s alpha = 0.79).

### 2.5. Fidelity Assessment

A fidelity form was developed using the OCD and anti-disgust cognitive behavioral manuals to assess therapists’ adherence to the treatment manual. Six items on the form assess four dimensions of fidelity: (a) session agenda, (b) rationale and treatment model, (c) psychoeducation and techniques offered, and (d) delivered homework. Two fidelity evaluators, both clinical psychologists, asked therapists to ensure that the interventions they used in sessions were appropriately tailored to the four dimensions of fidelity. Fidelity evaluators assigned a score to therapist responses ranging from 1 (not at all) to 5 (completely), indicating the extent to which the therapist followed the manual. Overall, 89% agreement was obtained between the two fidelity evaluators.

It is worth noting that most participants (*n* = 41; 78%) refused to have their voices recorded during the sessions. Eleven participants consented to record their voice sessions, resulting in 165 audio recordings. The agreement rate between therapists using these recorded voices was estimated to be 81%. Thus, no videotapes of the session exist, and the fidelity assessment was solely based on evaluators’ reports.

Moreover, the two therapists evaluated participants’ compliance with homework assignments using a Likert scale ranging from 0 (did not complete the assignment at all) to 5 (did the homework completely). Both groups adhered to their assigned tasks well (AD-CBT: M = 3.2; SD = 1.1; CBT: M = 3.1; SD = 1.3; t = 1.81; *p* > 0.06). In order to monitor or assess therapists’ ability to deliver treatments, participants in both groups were asked to rate their therapists’ treatment offerings. On average, AD-CBT and CBT participants scored 82% and 79% proficiency in delivering treatment, respectively.

### 2.6. Procedure

All participants (*n* = 60) were recruited via social media platforms (LinkedIn and Instagram) or newspaper advertisements. Following that, an initial evaluator (a clinical psychologist with a Ph.D. in clinical psychology) obtained informed consent and conducted clinical and diagnostic interviews in a mental health center while remaining blind to the study. He then referred eligible participants to another center’s research assistant. The authors separated the assessment center from the treatment center to minimize biases. Furthermore, a research assistant independently generated the random assignment sequence and assigned participants to the corresponding intervention arms using an automatic number generator. The participants were evaluated a total of four times: pretreatment (T0), prior to beginning ERP sessions (T1), posttreatment (T2), and a three-month follow-up (T3). The study scales were completed in a counterbalance style, accounting for sequence effects.

Following the conclusion of the last session, all participants were reassessed using all available measures. Three months later, participants were invited to the treatment center for the fourth time to complete the measures. Except for one participant in the CBT condition (Figure 1), all participants responded to the scales. The mentioned participant began taking new medication after experiencing severe OCD and depression symptoms during the three-month follow-up period.

### 2.7. Statistical Analysis

IBM SPSS software version 24.0 was used to analyze the data. Before conducting analyses, all fundamental assumptions of analysis of variance were obtained and validated, including normality, homogeneity of variance, and sphericity of the covariance matrix. Tabachnick and Fidell [49] state that participants with missing values greater than 5% can be omitted (*n* = 0). The analyses were conducted on participants in both groups who had completed baseline measurements at T0 (*n* = 26 participants in each group). We used mixed-design ANOVAs to compare T0 vs. T1 as the primary outcome and T0 vs. T2 and T0 vs. T3 as the secondary outcomes. In all mixed-design ANOVAs, Time (T0, T1, T2, and T3) was the within-subject factor, Group (AD-CBT and CBT) was the between-subjects factor, and DPSS, AS, and Y-BOCS were the dependent variables.

To determine whether the trajectories of outcome variables in AD-CBT and CBT differed between participants, a series of mixed models with DPSS, AS, and Y-BOCS scores as dependent variables and Group, Time (T0–T3), and the interaction between Group and Time as independent variables were applied (fixed effects). Finally, to determine whether the predominance of AD-CBT over CBT remains significant when depression (BDI-II) positive and negative affects (PANAS) are accounted for, we employed a mixed-design ANCOVA. Positive and negative affects were included as covariates in these analyses because they theoretically influence the effects of treatments on outcomes [20]. All analyses were performed with a random intercept.

## 3. Results

### 3.1. Demographic Characteristics

Data from participants who completed the baseline phase (T0) were included in the final analysis (*n* = 52; AD-CBT = 26; CBT = 26). They ranged in age from 19 to 40 years (M = 28.42, SD = 3.57 for AD-CBT; M = 27.96, SD = 3.47 for CBT). As shown in Table 2, there were no statistically significant differences in demographic characteristics between the two groups.

### 3.2. Primary Outcomes

The acceptance rate of ERP in AD-CBT and CBT groups was 97% and 85%, respectively. Intriguingly, none of the participants of either group dropped out during ERP sessions. Only one participant in the AD-CBT group (compared to four participants in the CBT group) refused to participate in the ERP sessions in T1.

The mixed-design ANOVAs was performed to determine total differences in both primary and secondary outcomes. The results revealed the overall significant main effects of Time and Time*Group interaction effects in all outcome variables. (1) ***DPSS*** (Time: Wilks λ = 0.12; F(3, 48) = 111.98, *p* < 0.001, η^2^ = 0.87, Time*Group: Wilks λ = 0.40; F(18, 334) = 23.56, *p* < 0.001, η^2^ = 0.60); (2) ***AS*** (Time: Wilks λ = 0.04; F(3, 48) = 385.22, *p* < 0.001, η^2^ = 0.96, Time*Group: Wilks λ = 0.32; F(3, 48) = 34.71, *p* < 0.001, η^2^ = 0.68); (3) ***Y-BOCS*** (Time: Wilks λ = 0.03; F(3, 48) = 490.42, *p* < 0.001, η^2^ = 0.97, Time*Group: Wilks λ = 0.56; F(3, 48) = 12.55, *p* < 0.001, η^2^ = 0.44) and (4). These differences require further investigation because they did not indicate which group differs from the other group in terms of the study variables. The results are summarized in Table 3.

**Figure 2:**
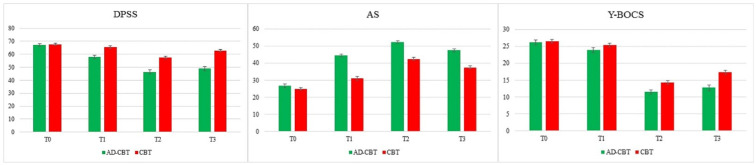
Longitudinal changes of primary outcomes for the two groups. **DPSS** = Disgust Propensity/Sensitivity Scale, **AS** = Acceptance Scale, **Y-BOCS** = Yale–Brown Obsessive Compulsive Scale, AD-CBT = Anti-Disgust Cognitive Behavioral Therapy; CBT = Cognitive Behavioral Therapy, **T0** = Pretreatment, **T1** = Before ERP sessions, **T2** = Posttreatment, **T3** = Three-month follow-up.

In all outcome variables, there were significant main effects of Time and Time*Group interactions. Next, to test whether the AD-CBT may lead to greater change in DPSS, AS, and Y-BOCS compared to the CBT alone, paired t-tests were conducted, which revealed significant changes in all outcome variables in both AD-CBT (DPSS t(25) = 9.37, *p* < 0.001, **Cohen’s** *d* = 1.54, **effect size** *r* = 0.61), (AS t(25) = −18.67, *p* < 0.001, **Cohen’s** *d* = 3.67, **effect size** *r* = 0.88), (Y-BOCS t(25) = 13.18, *p* < 0.001, **Cohen’s** *d* = 0.55, **effect size** *r* = 0.27), and CBT groups (DPSS t(25) = 5.08, *p* < 0.001, **Cohen’s** *d* = 0.39, **effect size** *r* = 0.19), (AS t(25) = −9.65, *p* < 0.001; **Cohen’s** *d* = 1.52, **effect size** *r* = 0.52), Y-BOCS t(25) = 5.51, *p* < 0.001, **Cohen’s** *d* = 0.37, **effect size** *r* = 0.18) from T0 (baseline) to T1 (before beginning ERP sessions). In summary, participants in both groups demonstrated decreased disgust propensity/sensitivity, OCD severity, and a greater capacity for non-judgmental acceptance of negative emotions, such as disgust in T1 compared to T0. The effect size and Cohen’s d indicated that these therapeutic changes occurred more frequently in the AD-CBT group. In other words, the results indicated that there were significant differences in primary outcomes between AD-CBT and CBT prior to the start of the ERP session (T1). The longitudinal changes in primary outcomes for each group are represented in Figure 2.

Direct pairwise comparisons (independent t-tests) were used to establish the superiority of AD-CBT over CBT at each time point (T0, T1, T2, T3). These results are presented in Table 4.

As shown in Table 4, there was no statistically significant difference between the two groups in the baseline. However, following the anti-disgust cognitive intervention, AD-CBT participants demonstrated significantly lower disgust propensity and sensitivity and increased emotion acceptance than CBT. There were no significant differences in OCD severity (Y-BOCS) between the two groups prior to ERP sessions (T1). However, AD-CBT was superior to CBT in all variables during posttreatment and the three-month follow-up.

### 3.3. Secondary Outcomes and Trajectories

Paired t-tests were used to investigate secondary outcomes and trajectories in AD-CBT and CBT in two groups from T0 to T2 and T0 to T3. The paired comparisons are presented in Table 5. Overall, both groups of participants demonstrated a significant increase in non-judgmental acceptance of negative emotions following treatment, and these therapeutic gains persisted three months later. Additionally, both groups experienced decreased OCD severity, disgust propensity, and sensitivity following treatment and at the three-month follow-up compared to pretreatment. The effect size and Cohen’s d indicated that participants in the AD-CBT group achieved lower DPSS and Y-BOCS scores and higher AS scores than those in the CBT group during posttreatment and three-month follow-up.

### 3.4. Controlling for Depression, Positive and Negative Affect

We used a series of mixed-design ANCOVAs to determine whether the superiority of AD-CBT over CBT remained significant after controlling for depression and positive and negative affect (see Table 6). After adjusting for covariates, mixed-design ANCOVAs revealed no significant differences in time between DPSS and AS. However, even after controlling for covariates, the Y-BOCS changes remained significant. Furthermore, group differences in all three variables remained significant. Finally, ANCOVAs using a mixed design revealed that all outcome variables had overall significant Time*Group interaction effects.

## 4. Discussion

This study aimed to examine the effects of augmenting CBT with an anti-disgust cognitive intervention on belief about disgust, OCD severity, and disgust propensity/sensitivity. The findings indicated that supplementing CBT with an anti-disgust cognitive intervention significantly reduces OCD severity, disgust propensity/sensitivity, and increases acceptance of internal aversive experiences such as disgust compared to CBT alone. Previous research has adequately addressed the therapeutic effects of cognitive and behavioral techniques on the outcomes of the current study [8,9,50,51]. Thus, in the Section 4, we focused on the benefits of augmenting CBT with an anti-disgust cognitive intervention.

The current study’s findings indicate that, in comparison to the CBT group, participants in the AD-CBT group demonstrated a higher rate of ERP acceptance prior to engaging in ERP sessions (96% vs. 84%, respectively). Unfortunately, the small sample size precludes the detection of a significant difference between the two groups. Despite this, our study’s refusal rate for ERP is comparable to that of Ong et al. [52] (16% for CBT). The study results show that the rate at which ERP sessions are entered is not optimal. Prior to being exposed to anxiety/disgust arousing stimuli during the ERP sessions, only the cognitive behavioral model of OCD was presented to the CBT group. Simultaneously, participants in the AD-CBT group were educated about the function of disgust and then educated on recognizing disgust as a false signal. This may result in a greater tolerance for disgust-related distress [53].

Prior to ERP sessions, all participants who underwent treatment (AD-CBT or CBT) perceived disgust as a less negative emotion, experienced less severe OCD, and accepted aversive emotions more non-judgmentally. Additionally, they were no longer as easily disgusted as they were before pretreatment in everyday experiences. These changes were more pronounced in the AD-CBT group. Only two sessions of anti-disgust cognitive intervention were required to significantly decrease disgust propensity and sensitivity while simultaneously increasing emotion acceptance in C-OCD individuals. However, neither AD-CBT nor CBT participants reported significant reductions in OCD severity prior to ERP sessions. These findings reaffirmed the critical nature of ERP in the treatment of OCD. Anti-disgust appears to have a more significant effect on OCD severity than CBT, as participants in the AD-CBT group had significantly less OCD severity at posttreatment and three-month follow-up than CBT.

The AD-CBT participants discovered that, often, the contamination feeling is spontaneously reduced [28]; as a result, they regarded disgust as a false alarm. Thus, providing more helpful information about disgust prior to the start of ERP sessions results in a greater acceptance of disgust and a decrease in OCD symptoms and disgust propensity/sensitivity. In line with the current findings, Abramowitz et al. [54] demonstrated that when C-OCD individuals experience intense emotions, they experience dysfunctional appraisal. Moreover, Fink et al. [50] reported that cognitive reinterpretation is associated with decreased disgust in patients with OCD.

The AD-CBT participants, but not the CBT participants, were given new information about distinct characteristics of disgust and contamination that fundamentally contradicts the information they already possessed (e.g., *a disgusting stimulus is not undoubtedly dirty and* vice versa, *or disgust feeling is not always an indication of real germs but also originates from how we responded to disgusting sensations in similar previous events*). This is consistent with Mathews and Mackintosh’s [55] assertion that the availability of new functional information results in adaptive evaluations in patients with OCD and anxiety disorders. Furthermore, when participants recognize disgust as a safe emotion, they see no reason to avoid situations that elicit disgust. Reduced disgust avoidance was observed to be significantly associated with the resolution of OCD symptoms [56].

The disgust response can also be explained by how confident OCD participants are in their ability to confront disgusting stimuli. According to Viol et al. [57], individuals with OCD do not trust their coping mechanisms when they are disgusted, in part due to hyperarousal. Anti-disgust experiments, such as “*hand in the honey*” or “*dirty chair*”, reminded AD-CBT participants that it is possible to experience a relatively intense disgust sensation despite the absence of a real threat or germs. Given the relationship between disgust sensitivity and OCD symptoms [58], it appears as though the experiments reduced disgust propensity and sensitivity first, and then, OCD symptoms. Individuals with OCD frequently exhibit disgust sensitivity or an ability to predict how aversive the disgust will be perceived. As a result, they forecast adverse events following exposure to repulsive stimuli [51]. Behavioral experiments allow for the examination of the prediction. Both groups demonstrated a significant decrease in their sensitivity to disgust. Thus, behavioral experiments are assumed to have sufficiently challenged the prediction. Furthermore, our findings indicated that separating physical and moral contamination in AD-CBT aided in enhancing behavioral experiment effects.

Additionally, the cognitive behavioral model of OCD emphasizes the destructive nature of certain beliefs associated with disgust [59]. Similarly, previous research has established that disgust results in an exaggeration of disgust-related threats [60]. Participants in both groups receive cognitive restructuring to address these beliefs, but those in the AD-CBT group also receive education on “*spontaneous regression*”. In the context of disgust-arousing events, this type of psychoeducation indirectly targets hyper-responsibility and overestimation of a threat [28]. Therefore, we can infer that cognitive intervention was more intense, diverse, and suited to disgust in the AD-CBT group.

Intriguingly, the results also demonstrated that, after adjusting for depressive symptoms, positive and negative affect, there is no significant difference in both groups’ acceptance of internal experiences, disgust propensity, and sensitivity from pretreatment to posttreatment and three-month follow-up. The current findings are most likely the result of a significant relationship between disgust and negative affect, or depression on the one hand [9,61,62] and strong associations between acceptance of internal events and negative affect on the other hand [25]. However, even after controlling for covariates, the changes in OCD severity from pretreatment to posttreatment and three-month follow-up were significant. Once more, when compared to CBT, the AD-CBT was associated with additional benefits.

### Limitations and Strengths

The current study established that disgust is amenable to cognitive intervention and provided additional justification for treating emotional beliefs in C-OCD. However, the results should be interpreted in light of several limitations. First, the sample size was small but adequate. Repeating the study with a larger sample is suggested. Second, recent research indicates that when people are disgusted, they exhibit distinct psychological and physiological responses [57]. In the current study, disgust is assessed solely through self-report. It is suggested that future research would benefit from a multidimensional assessment approach. Third, the majority of both groups’ participants were female. Although there were no gender differences between the two groups, given the effect of female hormones on disgust and OCD severity [63], excluding the menstrual period from our interpretations of the current findings limits our interpretations. Fourth, some research indicates that disgust sensitivity is more strongly associated with C-OCD than disgust propensity [58], implying that authors should investigate the effect of disgust-related interventions on these factors separately. Fifth, we limited comorbid conditions to major depressive disorder and anxiety disorders. Although more than two-thirds of individuals with OCD have one or more of these co-occurring disorders, they also have other psychiatric disorders [2]. Consequently, our findings are limited to OCD individuals with or without MDD and anxiety disorders. Finally, both groups received active treatment; a non-active treatment or waitlist control group is strongly recommended.

## 5. Conclusions

For more than a decade, the importance of addressing disgust in C-OCD has been emphasized [17]. Consistent with Cisler et al. [64], the current study discovered that addressing disgust-related cognitions in C-OCD increased the response to CBT while decreasing the ERP refusal rate. Unlike strategies such as counterconditioning, the present findings were obtained in the context of real and long-term disgusting stimuli that patients routinely confront in their daily lives. This study showed that a more constructive interpretation of disgust is associated with a decreased disgust response, less severe OCD, and a greater willingness to confront disgust-arousing stimuli. As well as the critical role of behavioral components (i.e., ERP), these results favor cognitive reconstructing in CBT.

## Figures and Tables

**Table 1:** Content summary of each AD-CBT and CBT session.

Session	AD-CBT	CBT
**1–2**	CBT Psychoeducation: introducing the CBT model; case formulation; learning the role of avoidant behaviors and the importance of ERP	CBT Psychoeducation: introducing the CBT model; case formulation; learning the role of avoidant behaviors and the importance of ERP
**3–4**	Anti-disgust cognitive intervention	Continued CBT Psychoeducation
**5–14**	Exposure and response prevention	Exposure and response prevention
**15**	Relapse prevention	Relapse prevention

Note: AD-CBT = Anti-Disgust Cognitive Behavioral Therapy; CBT = Cognitive Behavioral Therapy; ERP = Exposure and Response Prevention.

**Table 2:** Detailed demographic characteristics of the two groups.

Demographic	Level	AD-CBT (*n* = 26)		CBT (*n* = 26)		*χ^2^*/*t* (*df*)	*p*
		*N*	%	*N*	%		
**Gender**	Male	8	31	4	15	0.57 (1)	0.39
	Female	18	69	22	85		
**Age**	19–25	5	19	4	15	0.02 (3)	0.94
	26–30	10	38	12	46		
	31–35	7	27	6	23		
	36–40	4	15	4	15		
**Educational Levels**	Diploma	5	19	6	23	0.01 (2)	0.98
	Bachelor’s degree	15	58	16	61		
	High-level education	6	23	4	15		
**Comorbidity**	Yes	8	30	6	23	0.39 (1)	0.75
	No	18	70	20	77		
**Medications**	Used	12	46	10	38	0.31 (1)	0.57
	Not used	14	64	16	62		

**Table 3:** Mean and standard deviation of primary outcomes and results of mixed-design ANOVAs groups.

	AD-CBT (*n* = 26)				CBT (*n* = 26)				Results of Mixed-Design ANOVAs
	T0	T1	T2	T3	T0	T1	T2	T3	
	M (SD)	M (SD)	M (SD)	M (SD)	M (SD)	M (SD)	M (SD)	M (SD)	
**DPSS**	67.31 (5.13)	58.00 (6.82)	46.34 (8.07)	48.88 (8.63)	67.56 (4.63)	65.69 (4.88)	57.84 (5.36)	62.58 (5.09)	Time: F(1, 50) = 249.25, *p* < 0.001, η^2^ = 0.83 Group: F(1, 50) = 28.75, *p* < 0.001, η^2^ = 0.36 Time*Group: F(1, 50) = 59.16, *p* < 0.001, η^2^ = 0.54
**AS**	26.77 (5.38)	44.50 (4.17)	52.15 (3.94)	47.46 (4.43)	24.92 (4.23)	31.00 (5.69)	42.34 (4.71)	37.42 (5.32)	Time: F(1, 50) = 594.52, *p* < 0.001, η^2^ = 0.92 Group: F(1, 50) = 63.37, *p* < 0.001, η^2^ = 0.55 Time*Group: F(1, 50) = 18.44, *p* < 0.001, η^2^ = 0.33
**Y-BOCS**	26.15 (4.01)	23.91 (4.11)	11.46 (3.22)	12.77 (3.99)	26.53 (2.97)	25.42 (2.93)	14.31 (2.74)	17.27 (2.88)	Time: F(1, 50) = 1042.20, *p* < 0.001, η^2^ = 0.95 Group: F(1, 50) = 7.20, *p* < 0.01, η^2^ = 0.13 Time*Group: F(1, 50) = 23.32, *p* < 0.001, η^2^ = 0.32

**AD-CBT** = Anti-Disgust Cognitive Behavioral Therapy, **CBT** = Cognitive Behavioral Therapy, **DPSS** = Disgust Propensity/Sensitivity Scale, **AS** = Acceptance Scale, **Y-BOCS** = Yale–Brown Obsessive Compulsive Scale, **T0** = Pretreatment, **T1** = Before ERP sessions, **T2** = Posttreatment, **T3** = Three-month follow-up.

**Table 4:** Independent *t*-tests in AD-CBT and CBT to determine whether AD-CBT is superior to CBT.

	T0			T1			T2			T3		
	T (50)	Cohen’s *d*	Effect Size *r*	T (50)	Cohen’s *d*	Effect Size *r*	T (50)	Cohen’s *d*	Effect Size *r*	T (50)	Cohen’s *d*	Effect Size *r*
**DPSS**	−0.25	0.07	0.03	−4.67 ***	1.32	0.55	−6.05 ***	1.71	0.65	−6.97 ***	1.97	0.70
**AS**	1.37	0.39	0.19	9.77 ***	2.76	0.81	8.13 ***	2.30	0.75	7.39 ***	2.09	0.72
**Y-BOCS**	−0.39	0.11	0.05	−1.51	0.43	0.21	−3.43 **	0.97	0.44	−4.66 ***	1.32	0.55

**DPSS** = Disgust Propensity/Sensitivity Scale, **AS** = Acceptance Scale, **Y-BOCS** = Yale–Brown Obsessive Compulsive Scale, **T0** = Pretreatment, **T1** = Before ERP sessions, **T2** = Posttreatment, **T3** = Three-month follow-up. ** *p* < 0.01; *** *p* < 0.001.

**Table 5:** Paired t-tests in AD-CBT and CBT for secondary outcomes and trajectories.

	AD-CBT (*n* = 26)				CBT (*n* = 26)							
	T0 vs. T2		T0 vs. T3		T0 vs. T2				T0 vs. T3			
	T (25)	Cohen’s *d*	Effect Size *r*	T (25)	Cohen’s *d*	Effect Size *r*	T (25)	Cohen’s *d*	Effect Size *r*	T (25)	Cohen’s *d*	Effect Size *r*
**DPSS**	14.75 ***	3.10	0.84	12.67 ***	2.60	0.79	10.45 ***	1.94	0.70	6.82 ***	1.02	0.45
**AS**	−25.26 ***	5.38	0.94	−21.44 ***	4.23	0.90	−21.31 ***	3.89	0.89	−12.02 ***	2.60	0.79
**Y-BOCS**	31.94 ***	4.04	0.90	30.14 ***	3.34	0.86	23.33 ***	2.27	0.90	14.85 ***	3.16	0.84

**AD-CBT** = Anti-Disgust Cognitive Behavioral Therapy, **CBT** = Cognitive Behavioral Therapy, **DPSS** = Disgust Propensity/Sensitivity Scale, **AS** = Acceptance Scale, **Y-BOCS** = Yale–Brown Obsessive Compulsive Scale, **T0** = Pretreatment, **T2** = Posttreatment, **T3** = Three-month follow-up. *** *p* < 0.001.

**Table 6:** Results of mixed-design ANCOVAs controlling for depression, positive and negative affect.

	Covariates									Outcome Variable
	Depression			Positive Affect			Negative Affect			
	F(1, 47)	*p*	η^2^	F(1, 47)	*p*	η^2^	F(1, 47)	*p*	η^2^	
**DPSS**	1.65	0.20	0.03	2.77	0.10	0.05	0.31	0.58	0.01	Time: F(1, 47) = 3.63, *p* = 0.06, η^2^ = 0.07 Group: F(1, 47) = 29.45, *p* < 0.001, η^2^ = 0.38 Time*Group: F(1, 47) = 65.56, *p* < 0.001, η^2^ = 0.58
**AS**	2.47	0.12	0.05	0.33	0.57	0.01	2.54	0.12	0.05	Time: F(1, 47) = 1.61, *p* = 0.21, η^2^ = 0.03 Group: F(1, 47) = 59.83, *p* < 0.001, η^2^ = 0.56 Time*Group: F(1, 47) = 27.75, *p* < 0.001, η^2^ = 0.33
**Y-BOCS**	0.06	0.80	0.001	1.64	0.21	0.03	1.06	0.31	0.02	Time: F(1, 47) = 10.83, *p* < 0.001, η^2^ = 0.19 Group: F(1, 47) = 8.26, *p* < 0.01, η^2^ = 0.15 Time*Group: F(1, 47) = 20.70, *p* < 0.001, η^2^ = 0.31

**DPSS** = Disgust Propensity/Sensitivity Scale, **AS** = Acceptance Subscales, **Y-BOCS** = Yale–Brown Obsessive Compulsive Scale.

## Data Availability

Data can be made available from the first author upon request.
